# “The Government Is Keeping Me From My Mother”: Impacts of Visitation Restrictions as Reported by Family Members

**DOI:** 10.1093/geroni/igaf122.1945

**Published:** 2025-12-31

**Authors:** Jennifer Heston-Mullins, Lirisha Tuladhar

**Affiliations:** Miami University, Oxford, Ohio, United States; Miami University, Oxford, Ohio, United States

## Abstract

The Ohio Nursing Home Family Satisfaction Survey (FSS) is a self-administered mailed questionnaire with an online option conducted every two years per state mandate. Data collection for the tenth implementation of the FSS occurred between August 2021 and May 2022, while some nursing facilities in the state were still restricting visitation based on occurrences of COVID-19 infections. While the 2021-22 FSS questionnaire did not ask about family members’ experiences with visitation restrictions during COVID-19, respondents were provided the opportunity to leave general comments at the end of the survey and to participate in a separate, online COVID-19 survey where they could provide input about their experiences during the pandemic. A total of 13,010 family members of nursing facility residents responded to the 2021-22 FSS and 764 nursing home family members responded to the COVID-19 survey. Using content analysis, researchers from Scripps Gerontology Center at Miami University analyzed 2,890 open-ended comments from the FSS and COVID-19 surveys and identified 269 comments pertaining to visitation restrictions, which were then coded and analyzed for themes. While some family members reported appreciation or neutrality regarding visitation restrictions, the majority of commenters reported negative social and emotional impacts on both residents and family members and negative physical and cognitive impacts on residents, particularly those living with dementia. This session will share findings from the qualitative analysis and considerations for policy makers in the event of future infectious disease outbreaks.

